# TACI Isoforms Regulate Ligand Binding and Receptor Function

**DOI:** 10.3389/fimmu.2018.02125

**Published:** 2018-10-02

**Authors:** Yolanda Garcia-Carmona, Adrian T. Ting, Lin Radigan, Sai Krishna Athuluri Divakar, Jose Chavez, Eric Meffre, Andrea Cerutti, Charlotte Cunningham-Rundles

**Affiliations:** ^1^Department of Clinical Immunology, Precision Immunology Institute, Icahn School of Medicine at Mount Sinai, New York, NY, United States; ^2^Department of Oncological Sciences, Icahn School of Medicine at Mount Sinai, New York, NY, United States; ^3^Department of Immunobiology, Yale University School of Medicine, New Haven, CT, United States; ^4^Catalan Institute for Research and Advance Studies (ICREA), Barcelona, Spain; ^5^Program for Inflammatory and Cardiovascular Disorders, Institut Hospital del Mar d'Investigacions Mèdiques (IMIM), Barcelona, Spain; ^6^Department of Medicine and Pediatrics, Icahn School of Medicine at Mount Sinai, New York, NY, United States

**Keywords:** TACI, isoforms, B cell, activation, TLR9

## Abstract

TACI signals activate B cell proliferation, isotype switch and antibody production in both normal immunity and autoimmune states. In contrast to murine TACI, the human TACI gene undergoes alternative splicing to produce short and long isoforms (TACI-S and TACI-L). In previous studies, we showed that transduction of the short, but not long isoform, into murine B cells or human pre-B cells lacking TACI, caused them to become transcriptional and morphologically identical to plasma cells. These data suggest that the expression of different isoforms in humans provides unique controls on B cell maturation. In these studies we show that TACI-S and TACI-L form complexes in a ligand-independent manner, not dependent on a single extracellular domain. Both TACI isoforms are detectable in the endosomal cellular compartment where they co-localize with MyD88, TRAF6, and the activated 65 kDa form of TLR9, depending on a conserved intracellular TACI sequence. In contrast to TACI-L expressing cells, or cells bearing both isoforms, TACI-S binds ligands BAFF and APRIL with substantially greater affinity and promotes enhanced NF-kB activation. Using isoform-specific monoclonal antibodies, we show that while TACI-L is predominant as a surface receptor surface on human B cells, significantly more TACI-S is noted in the intracellular compartment and also in marginal zone, isotype switched and plasmablast in resting B cells. TACI-S is increased in tonsillar B cells and also in the intracellular compartment of activated peripheral B cells. These data shows that alternative splicing of the human TACI gene leads to two isoforms both of which intersect with MyD88 and TRAF6 and form complexes with TLR9, but the two isoforms have different ligand binding capacities, subcellular locations and activation capabilities.

## Introduction

Transmembrane activator calcium modulator and cyclophilin ligand interactor (TACI) is a receptor expressed on B cells, especially CD27^+^ marginal zone, memory B cells, and plasma cells (1, 2). Activation of TACI by its ligands, a proliferation inducing ligand (APRIL) and B cell-activating factor (BAFF), promotes up-regulation of activation induced cytidine deaminase (*AICDA*) mRNA, isotype switch and B cell maturation to T-cell independent antibody production (2, 3). While TACI activation also induces B cell proliferation, isotype switch and terminal differentiation in mouse models, these signals can also limit clonal expansion by promoting up-regulation of *Blimp1* mRNA, which fosters terminal plasma cell differentiation (4, 5). In humans, loss of one allele of the gene encoding TACI (*TNFRSF13B*) in the Smith-Magenis genetic syndrome, revealed that TACI haplo-insufficiency decreased TACI expression on memory B cells, and resulted in impaired B cell activation and antibody secretion (6, 7). Reinforcing the role of TACI as a B cell activator, missense mutations in this receptor lead to loss of B cell function and impaired repertoire selection in about 8% of humans with common variable immune deficiency (CVID) (2, 8–11). While mutation bearing relatives are not commonly hypogammaglobulinemic, their B cells fail to upregulate *AICDA* mRNA when activated by TACI agonists and display defective central B cell tolerance, revealing both intrinsic and dominant immune defects (9, 10).

Dissecting the complex biology of this receptor has gained much from study of murine models; however, in contrast to the murine *Tnfrsf13b* gene with two ligand-binding domains, the human *TNFRSF13B* gene has an additional 5′ exon which encodes a short terminal sequence. This permits skipping of exon 2 containing the first cysteine rich domain (CRD1), leading to the production of a second, shorter isoform lacking the first ligand-binding domain (12). As both isoforms are expressed in human B cells, we previously examined the functional differences between isoforms transduced into murine and human B cells lacking TACI expression. While murine A20 B cells and even more dramatically, human NALM6 pre-B cells transduced with the long TACI isoform (TACI-L), retained surface CD19 and IgG, cells transduced with the short isoform (TACI-S) lost these B cell characteristics and gained both transcriptional and morphologic features of plasma cells (13). The current study examines the structural requirements for receptor assembly, differential ligand binding and activation of these isoforms, the impact of TACI missense mutations, and the intracellular associations of TACI isoforms with MyD88, TRAF6, and TLR9. We investigated also the distribution of TACI in human B cell populations and how activation affects TACI isoform expression in human B cells.

## Methods

### TACI receptor assembly examined by FRET and co-immuno-precipitation

As human B cells populations generally contain mRNA and protein for both isoforms (13), we examined the complexes formed by TACI-S and/or TACI-L after transfection into (HEK) 293T cells (ATCC), using fluorescence resonance energy transfer (FRET) (14). For this, TACI-L and TACI-S cDNA were amplified by PCR, labeled with mCherry or YFP (Primers are listed on Supplemental Table SI), and cloned into the pCINeo mammalian expression vector (Promega). Human TACI mCherry labeled mutants, C104R, A181E, and S194X, were also generated in each isoform using QuikChange II XL Site-Directed Mutagenesis Kit (Agilent) (Primers are listed on Supplemental Table SI). As a control, plasmid pReceiver-huCD40-eYFP was obtained from Genecopoeia. For FRET analyses, HEK-293T cells were transiently co-transfected with 1 μg of each YFP and mCherry plasmid pair (TACI-L, TACI-S, or CD40) using SuperFect reagent (Qiagen). After 48 h incubation at 37°C in 5% CO2 in DMEM medium (Gibco) with 10% FBS, transfected cells were washed, suspended to 500,000 cells/ml and FRET signals determined by FACS (LSRII or LSRFortessa, BD Biosciences). Similarly, TACI-L with C104R, A181E, and S194X mutations found in CVID subjects, were examined, pairing these with TACI-S or as a control, CD40-eYFP. For all transfected cells, both YFP and mCherry expression were determined (see Supplemental Figure S1). Data were analyzed using FlowJo software (Tree Star, Inc.).

To determine the effects of adding ligands on the FRET signal, rhAPRIL (0, 20, 100, or 200 ng) or rhBAFF (0, 5, 10, 20, or 50 ng) (R&D Systems) were added and samples were analyzed at different time-points (0, 2, 10, and 30 min). For validation of complexes found in FRET studies by immunoprecipitation, we then generated the constructs FLAG-TACI-L and hemagglutinin labeled (HA) TACI-L, TACI-S, and the selected mutants C104R, A181E, or S194X (primers are listed on Supplemental Table SI). These constructs were subsequently cloned into pcDNA3.1(+) plasmid, using In-Fusion system (Clontech), following manufacturer's instructions. HEK-293T cells were transfected as described above. After 48 h, cells were harvested and total cell lysates were incubated overnight at 4°C with 15 μl of anti-FLAG M2 magnetic beads (Sigma-Aldrich). Twelve hours later, immune complexes were precipitated and beads were incubated for 1 h with 30 μg of FLAG peptide (300 μg/ml) on ice and immunoprecipitated proteins were separated from the beads by centrifugation using Spin cups-cellulose acetate filters (Pierce). Immunoprecipitated proteins were separated by 4–12% SDS-PAGE and analyzed by immunoblot for the HA tagged proteins (Thermo-Fisher).

### Comparing isoforms and mutants in NF-Kappa B activation

To validate that functional TACI receptor complexes were produced and compare the signaling of TACI isoforms in transfected cells, we examined NF-kB activation after transfection of TACI-S or TACI-L into HEK-293T cells (or for comparison, the missense mutants C104R, A181E, or S194X), along with 0.8 μg of NF-kB luc reporter and 0.5 μg control pRL-null plasmids. Cells were cultured for 48 h after transfection to allow full TACI expression, and examined with and without activation for 6 h with 100 ng/ml rhAPRIL or rhBAFF. Equal amounts of DNA (0.8 μg) were used for each transfection, using empty vector DNA as needed. Reporter gene activity was determined after 48 h and NF-kB luciferase induction was normalized to Renilla luciferase intensity. As both FRET and immune precipitation studies suggested that TACI-S and TACI-L could form complexes with each other, we then tested NF-kB luciferase induction in HEK-293 cells transfected with both isoforms, validating the presence of each by the different fluorescent probes. For these studies, the values reported represent Relative Luciferase Activity (RLA) and are the mean ± SD from 5 to 7 independent experiments.

### TACI receptor assembly

As TACI-S lacks the CRD1 but is clearly functionally active, this domain does not appear to be needed for TACI assembly, as previously suggested (15, 16). For this reason, to examine the requirements for TACI receptor complex, a set of extracellular domain deletion mutants were produced by PCR: these include (1) excision of the CRD1 (a construct equivalent to TACI-S), (2) construct with removal of exon 1 and CRD1 (ΔE1-CRD1), (3) the second cysteine rich domain (CRD2) (ΔCDR2), (4) both CRD1 and CRD2 (ΔCDR1-ΔCRD2; aa 21-104), and (5) the remaining extracellular 59 amino acids (Δaa105-164) (Primers for each are listed in Supplemental Table SII). Each of these constructs were also labeled with HA, and examined for co-precipitation with FLAG-TACI-L after transient transfection in HEK-293T cells as described above.

### TACI isoforms and mutants in B cells

To examine TACI isoforms in human B cells, the HA tagged human TACI-L and TACI-S GFP-constructs were stably transduced into the human B cell line BJAB, which lacks the endogenous expression of TACI (17). GFP positive cells were isolated by sorting (FACSAria II, BD Biosciences), expanded and maintained as previously described (13).

### Differential ligand binding for isoforms

As we had found that human TACI-S exhibits greatly enhanced activation in both transduced murine and human B cells (13), we tested if TACI-S might have an enhanced affinity for ligands as compared to TACI-L, which would provide a potential reason for these signaling differences. For this we separately incubated BJAB or HEK-293T cells bearing TACI-L or TACI-S (or both) with increasing amounts of FLAG-tagged multimerized MegaAPRIL (Adipogen) or MegaBAFF (Enzo) (0–800 ng). After this, 1 μg/mL biotin-anti-FLAG monoclonal M2 antibody was added to multimerize the ligands (18). Cells were washed, streptavidin-phycoerythrin-PE (Becton Dickinson) added and then examined by FACS (LSRII) (10, 13). To ensure comparable expression of TACI receptors on BJAB cells, GFP expression, integral to both constructs, was determined (13). For HEK-293T cells, comparable TACI expression was determined by FACS using an anti-TACI antibody which binds both TACI isoforms (mAb174, R&D). As our FRET studies suggested that mixed short and long isoforms may occur on human B cells, we also compared APRIL binding on BJAB and HEK-293T cells transduced with both TACI-S and TACI-L, separately labeled with either mCherry or YFP to validate transduction of both isoforms.

### Comparing ligand affinity of isoforms using microscale thermophoresis

As TACI-S isoform transfected cells had a greater affinity for ligands than TACI-L cells in binding assays, we then turned to a cell free system to quantify these differences. For this, FLAG-TACI isoform proteins were expressed in HEK-293T cells and purified with anti-FLAG beads. Highly purified recombinant TACI-L and TACI-S were then fluorescently labeled at their N-termini for microscale thermophoresis (MST) using the Monolith NT Protein Labeling Kit RED-NHS (NanoTemper Technologies, München, Germany). Briefly, proteins at concentrations of 20 μM were incubated with 4X dye at a ratio of 1:1 in labeling buffer. To determine the *K*d values of TACI-L and TACI-S for BAFF or APRIL, 100 nM of labeled FLAG-tagged TACI-L and TACI-S proteins were incubated with increasing concentrations (from 0 to 10^4^ nM) of BAFF or Mega-APRIL (Adipogen), for 30 min at room temperature in binding buffer (PBS) (17). The samples were then centrifuged, loaded into premium-coated capillaries (NanoTemper Technologies) and fluorescence values from the binding reactions determined using the Monolith NT.115 (Nano Temper Technologies). Binding data was analyzed using Affinity analysis software (NanoTemper) to determine the *K*d values for each TACI isoform for BAFF and APRIL.

### Endosomal expression of TACI isoforms

TACI is best known as a surface receptor, but it is assembled in the ER where it intersects with MyD88 along with other adaptors such as TNFR-associated factor 6 (TRAF6), important for NF-kB activation (15, 19, 20). To examine MyD88 and TRAF6 recruitment to TACI-S and TACI-L, isoforms labeled with mCherry were transfected into HEK-293T cells, grown on coverslips in 24-well multiwell plates for 48 h. Cells were stained with rabbit Ab to MyD88 (Millipore) or TRAF6 (Santa Cruz) and Alexa Fluor 647-conjugated secondary antibody (ThermoFisher Scientific), and nuclei were stained with 4′,6-diamidine-2′-phenylindole dihydrochloride (DAPI) (Boehringer Mannheim). In other experiments, BJAB cells were stained goat Ab to TACI (Santa Cruz). To identify the endocytic compartment, cells were incubated with transferrin-Alexa Fluor 647-conjugated (40 μg/ml Tfn-647, Molecular Probes) at 37°C for varying times 1–60 min, terminating the reaction by washing cells with ice-cold PBS and fixation with 4% paraformaldehyde in PBS. In other experiments, in order to further identify the late endosomal compartment, fixed transfected cells were stained with mAb Rab7 (Abcam). As negative controls, cells were co-transfected with plasmid pLVX-IRES-mCherry. All slides were cover-slipped with FluorSave reagent (Calbiochem) and analyzed by laser scanning confocal microscopy (Leica SP5 DMI). Further image processing was performed using Adobe Photoshop.

### TACI isoforms and intersection with TLR9

Our previous experiments demonstrated functional synergies between TACI and TLR9, including the shared requirement for MyD88 (20) and the observation that TACI and the activated 65 kDa form of TRL9 formed complexes in human B cells (9, 21). To examine the cytoplasmic intersection of TACI isoforms with TLR9 by confocal microscopy, we transfected HEK-293T cells with mCherry labeled TACI-S or TACI-L, or for comparison the S194X mutant, along with TLR9-YFP (pcDNA3-TLR9-YFP was a gift from Doug Golenbock; Addgene plasmid # 13642). We also examined complexes between TACI isoforms and TLR9 in FRET experiments in these cells as described above, using the S194X truncation TACI mutant as a control. We further examined TACI and TLR9 complexes by immunoprecipitation from BJAB cells, stably transduced with either TACI-S or TACI-L. For this, isoform-transduced BJAB cells were lysed, incubated with 1 μg biotin-labeled polyclonal anti-TACI (PepProtech) and then streptavidin Sepharose beads (GE Healthcare). In some experiments BJAB cells were incubated with rhAPRIL (200 ng/ml), ODN (3 μg/ml) or the combination of both, to gauge the effect of activation on the association of TLR9 with TACI isoforms. After washing the beads, adherent complexes were examined after PAGE, followed by blotted with polyclonal anti-TLR9 antibody (1:250, Abcam) and developed with anti-rabbit-HRP substrate (1:500 Thermo Fisher). For demonstrating the input for BJAB experiments, whole cell lysates were also examined, blotting separately for TACI and TLR9.

### Monoclonal antibodies for TACI isoforms

To distinguish TACI isoforms in human B cells, mAbs were then raised to the TACI-S isoform, using the junctional TACI segment GRSRVDQEERWSLSCRKEQGKFYD unique to this isoform. Monoclonal antibody candidates were then screened for specificity by FACS (LSRII), on isoform-bearing BJAB cells. Clone 2H12 was selected and the IgG2a antibody was purified from hybridoma cell supernatants by FPLC (Akta Purifier FPLC system, GE Healthcare). The commercial mAb (clone 11H3, eBiosciences) was found to bind only TACI-L bearing BJAB cells (**Figure 7A**). The specificity of these mAbs was further validated by MST as described above, in this case incubating from 0 to 10^4^ nM concentrations mAb with 100 nM fluorescently labeled recombinant FLAG-tagged TACI-L or TACI-S proteins for 30 min at room temperature in binding buffer (PBS) (17). The samples were then processed as described above to determine the *K*d values for mAbs 2H12 and 11H3 for each TACI isoform.

### Expression of TACI isoforms on human B cell populations

Peripheral blood was obtained from four normal adult donors. Tonsils were obtained from adult patients with follicular hyperplasia. The use of blood and tissue samples was approved by the Institutional Review Board of Mount Sinai School of Medicine (IRB). Human tonsillar mononuclear cells were obtained from fresh samples by mechanical disruption followed by separation on a Ficoll-Hypaque gradient; buffy coats from normal blood donors were also obtained from the New York Blood Center. Mononuclear cells (PBMCs) were isolated by Ficoll-Hypaque (Pharmacia, Uppsala, Sweden). To examine the expression of TACI isoforms on B cell subpopulations, B cells isolated by negative selection (CD19+) were stained with isoform specific mAbs in PBS with 1% BSA for 30 min at 4°C. Cells were subsequently washed and fixed with a 2% paraformaldehyde/PBS solution before analysis on BD LSRFortessa. For intracellular staining, cells were permeabilized after fixation for 20 min with BD FACS Permeabilizing solution 2 (BD), washed and stained with mAb TACI-L or mAb TACI-S. For indirect staining, cells were incubated with anti-mouse Alexa Fluor 488 (Invitrogen) using an IgG2a or IgG2b isotype control to validate the specificity of the staining. Living cells were identified by forward scatter and side scatter gating, and/or exclusion of 7-aminoactinomycin-D (eBioscience). B cell populations were discriminated by using CD19 APC-Cy7 (eBiosciences), CD27 PE (BD), CD38 Brilliant Blue, (eBiosciences), IgM APC (BD), IgD Pacific Blue (eBiosciences), CD20 FITC (BD), to designate naïve (IgD+CD27−), transitional (IgM+CD38+), marginal zone (IgD+CD27+), switched memory (IgD-CD27+), and plasmablasts (CD20^lo/−^CD38^hi^). To examine the effect of activation in TACI isoforms expression, mononuclear cells (PBMCs) were cultured for 4 days in the presence of ODN (3 μg/ml), CD40L/IL21 (200 and 20 ng/ml) (Invivogen) (R&D Systems) or anti IgM (10 μg/ml) (SouthernBiotech). Flow cytometry data analysis was performed using FlowJo data analysis software (Tree Star, Ashland, OR). As permeabilized cell staining results also include contributions from surface staining, we calculated the percentage of TACI surface and intracellular expression as previously described (22).

### Statistical analysis

Statistical analyses were performed using GraphPad Prism v.6 (GraphPad Software Inc., San Diego, CA). Values were expressed as mean ± standard error of the mean (SEM) or mean ± standard deviation (SD). Statistical significance was assessed by a two-tailed paired Student's *t*-test.

## Results

### Characterization of TACI hybrid complexes

**Figure 1:**
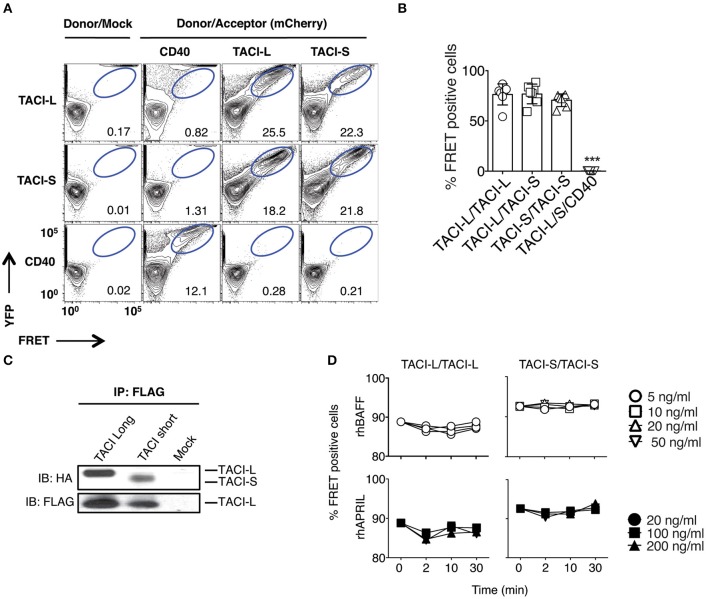
TACI Isoforms form hybrid complexes detected by FRET. **(A)** To examine isoform complexes, YFP and mCherry labeled TACI-L, TACI-S, and/or CD40-eYFP as a control, were co-transfected into HEK-293T cells. After 48 h, the molecular association was analyzed by FRET using FACS (LSRII). A minimum of 50,000 positive cells were examined in all experiments. The dot plot results in each panel, are shown for one sample, representative of the results for 6 different experiments. The numerical data given in each panel, are the percent of FRET positive cells, of the live cells in the gate, averaged for all 6 experiments. **(B)** Frequency of FRET positive cells in the double positive YFP and mCherry gate from **(A)**. Graph shows the mean ± SD. ***p < 0.001, two-tailed paired Student *t*-test of 6 independent experiments. **(C)** Either HA-labeled TACI-S or TACI-L transfected into HEK-293T cells, could be precipitated with TACI-L-FLAG as demonstrated in immuno-blotting using anti-HA staining. Lower panel shows FLAG expression as control. **(D)** Addition of increasing amounts of ligands BAFF or APRIL to the TACI-S and TACI-L complexes had no effect on the FRET signal, indicating that receptor assembly in these cells is ligand independent. Data are the mean ± SD from 3 independent experiments.

Previous work on the murine TACI receptor suggested that the CRD1 region was essential for receptor assembly (15). However, human TACI-S lacks the CRD1 domain entirely and exerts more intense NF-kB activation than TACI-L in both human and murine B cells (13), demonstrating that this domain is not essential for a functional TACI receptor. To examine isoform complex formation, mCherry labeled human TACI-S and YFP labeled TACI-L were both transfected into HEK-293T cells and TACI receptor assembly was examined by FRET. TACI-S and TACI-L produced complexes, not only with each other but also with the other isoform, whereas neither isoform assembled in complexes with YFP-CD40L (Figures 1A,B; see also Supplemental Figure S1 for the expression of YFP and mCherry for cells examined). Validating the possibility of mixed isoform complexes, transfected TACI-L-FLAG was able to precipitate either HA-labeled TACI-S or TACI-L (Figure 1C). TACI receptor assembly was ligand-independent, since addition of various concentrations of rhBAFF or rhAPRIL to either TACI-S and TACI-L transfected cells, had no effect on FRET signals (Figure 1D).

### Impact of TACI mutations on receptor assembly and function

**Figure 2:**
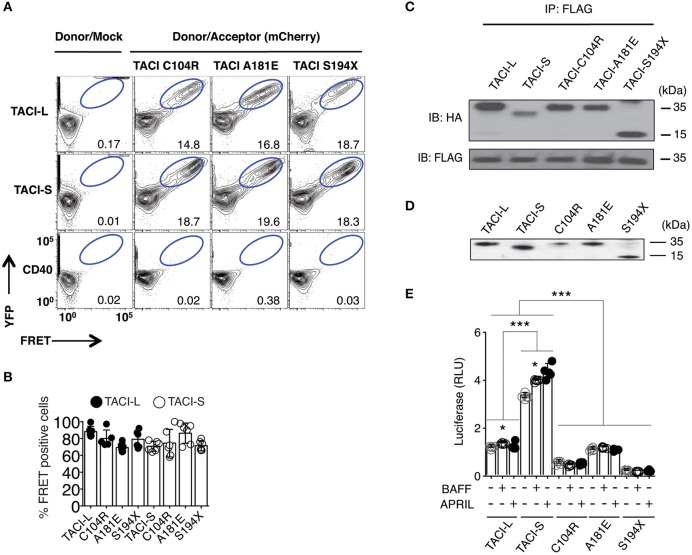
TACI variants with missense mutations bind un-mutated isoforms but lack signaling function. **(A)** TACI mCherry labeled mutants, C104R, A181E, and S194X were generated in the TACI-L and TACI-S isoforms, and co-transfected into HEK**-**293T cells with WT TACI-YFP. These were examined in FRET experiments by FACS (LSRII) to judge complex formation using CD40-eYFP as a control. The dot plot results in each panel, are shown for one sample, representative of the results for 6 different experiments. The numerical data given in each panel, are the percent of FRET positive cells, of the live cells in the gate, averaged for all 6 experiments. **(B)** Frequency of FRET positive cells in the double positive YFP and mCherry gate. Data shows average ± SD from 6 independent experiments. In other experiments, ligands, APRIL, or BAFF (0, 5, 20, or 50 ng/ml) were added to judge the effects on FRET signal, showing no alteration in the signal (not shown). **(C)** For validation of complexes found in FRET, complexes forming with FLAG-TACI were precipitated with anti-FLAG sepharose beads and run on 10% PAGE gels; immunoblots were developed with an anti-HA antibody. Lower panel shows FLAG expression control. **(D)** To examine NF-kB luciferase induction, TACI-S, TACI-L or mutant C104R, A181E and S194X constructs were transfected into HEK-293T cells, along with NF-kB–luc reporter and control pRL-null plasmids and cultured for 48 h; TACI expression was confirmed by western blot. **(E)** These cells were cultured with or without activation for 6 h with 100 ng/ml APRIL or BAFF. Reporter gene activity was determined, and NF-kB luciferase induction normalized to Renilla luciferase. Values reported are represented as Relative Luciferase Activity (RLA) and are the mean ± SD from 5 to 7 independent experiments. *p < 0.05; ***p < 0.001, two-tailed paired Student *t*-test. Western blot shows that all constructions are expressed.

As B cells of some CVID subjects carry heterozygous *TNFRSF13B* mutations, we then examined complexes in HEK-293T cells transfected with either mCherry-labeled TACI-S or TACI-L, along with either isoform containing missense mutations found in CVID (C104R, A181E, or S194X) or YFP-CD40L as control. As the FRET signal was preserved for each combination (Figures 2A,B) (but not for CD40), and each mutant could be co-precipitated with transfected FLAG-TACI-L from these cells, we conclude that these missense alterations do not disrupt receptor formation (Figure 2C). However, to test the functions of these receptors, we then analyzed NF-kB activation induced by TACI-L and TACI-S, as compared to TACI mutants C104R, A181E, and S194X, each transfected into HEK-293T cells with the expression of each verified by western blot (Figure 2D). Consistent with our previous data in transduced cells (13, 20), competent TACI receptors have a high baseline luciferase induction in the absence of ligand; however, TACI-S was more efficient in luciferase induction than TACI-L, both with and without ligand exposure (Figure 2E). In contrast, TACI variants with missense mutations displayed impaired or no function, as anticipated (15, 23) (Figure 2E).

### Assessment of TACI domains required for receptor assembly

**Figure 3:**
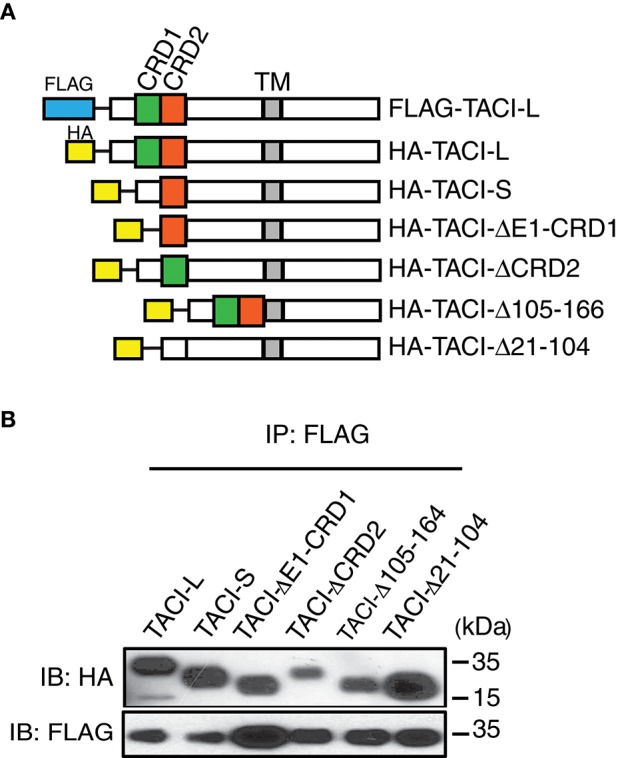
**(A)** TACI variants with diverse deleted extracellular domains retain capacity for complex assembly. Upper panel shows HA labeled-TACI deletion mutant products: Full length TACI-L, TACI-S (minus CRD1), exon 1 and CRD1 (ΔE1-CRD1), CRD2 (ΔCDR2), minus the remaining extracellular 59 amino acids (Δaa105-164) and excision of both CRD1 and CRD2 (Δ21-104) were constructed. **(B)** Constructs were transfected into HEK-293T cells along with TACI-L-FLAG; after cell lysis, precipitates were harvested with anti-FLAG sepharose beads, and complexes in lysates examined after PAGE and immunoblotting using an anti-HA antibody. FLAG expression was tested as control (lower panel). Data are representative of 3 independent experiments.

As the CRD1 was apparently not required for receptor function, we then sought other regions essential for assembly, using a panel of TACI constructs with deletions in selected domains: exon 1 and CRD1, CRD2 (the second ligand binding domain), the 79 extracellular amino acids linking the CRD2 to the transmembrane domain (Δ105-164), or both CRD1 and CRD2 (Δ21-104) (Figure 3A). In each case, FLAG-labeled TACI-L was able to co-precipitate TACI-S (lacking the CRD1), as well as all of the other tested TACI deletion variants (Figure 3B). This observations suggest that oligomerization of human TACI is likely to depend upon a number of extracellular (or transmembrane) associations, but is not limited to a single extracellular region.

### TACI-S displays increased ligand binding compared to TACI-L

**Figure 4:**
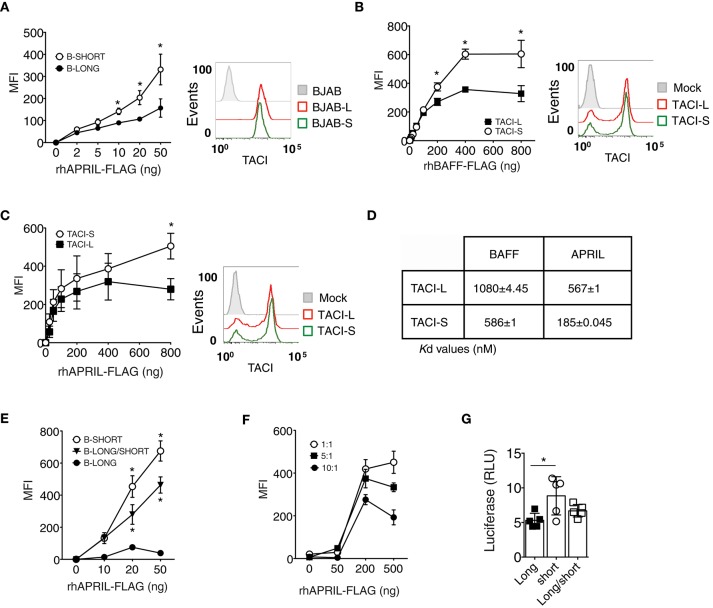
TACI-S display increased ligand binding. **(A)** To compare ligand binding capacities of isoforms, varying concentrations of FLAG-APRIL (0–50 ng/ml) were incubated with TACI-L or TACI-S transduced BJAB cells. Ligand binding was determined by FACS (LSRII) on washed cells, after incubation with anti-FLAG-PE. Comparable expression of TACI was determined by FACS (LSRII) using GFP expression. **(B,C)** To compare ligand binding in TACI-L or TACI-S transfected HEK-293T cells, cells were incubated with increasing amounts (0-800 ng) of either FLAG-BAFF **(B)** or FLAG-APRIL **(C)**. Comparable expression of TACI isoforms in each case was determined by FACS (LSRII) using anti-TACI antibody. **(D)** The binding affinity of TACI-L and TACI-S to ligands BAFF and APRIL was determined by Microscale thermophoresis (MST). The change in the thermophoretic signal produces these *K*d values (nM). Data represent the mean and SD of three independent thermophoresis measurements. **(E)** BJAB cells transduced with TACI-S and TACI-L, displayed binding of FLAG-APRIL intermediate between the TACI-S and TACI-L. **(F)** Diminishing ligand binding was also found for TACI-S HEK-293T cells also transfected with increasing ratios of TACI-L (ratios 1:1, 5:1, or 10:1) and incubated with to 0–500 ng of FLAG-APRIL. **(G)** HEK-293T cells transfected with both TACI-S and TACI-L, demonstrated intermediate NF-kB luciferase induction as compared to cells with either isoform. Values reported are represented as Mean of Fluorescence Intensity (MFI) **(A-C,E,F)** and as Relative Luciferase Units (RLU) **(G)** and are the mean ± SEM from 5 to 7 independent experiments. *p < 0.05, two-tailed paired Student *t*-test.

A previous study suggested that the CRD1 of human TACI-L contains a weaker ligand binding domain as compared to the second ligand binding region retained in the TACI-S isoform (24), potentially accounting for the more robust signaling of the TACI-S receptor we observed here. We tested the ability of TACI-L and TACI-S to bind to APRIL by incubating BJAB cells transduced with either TACI isoform with APRIL-FLAG. While both TACI isoforms had similar surface receptor expression (as shown) TACI-S cells exhibited greater APRIL binding over a wide range of ligand concentrations than TACI-L cells (Figure 4A). In addition, TACI-S transfected HEK-293T cells also showed enhanced binding for BAFF (Figure 4B) and APRIL (Figure 4C), as compared to TACI-L cells. To further quantify the binding affinity of TACI-L and TACI-S to ligands BAFF and APRIL, we also used microscale thermophoresis (MST) (25). As expected, the TACI-L isoform demonstrated a much weaker affinity for both BAFF and APRIL (a higher *Kd* for both) than TACI-S (Figure 4D).

As human B cells might bear TACI receptors of either or both isoforms, or as FRET experiments suggested, perhaps even receptors of mixed isoform content, we then examined BJAB cells transduced with both isoforms. As expected, these mixed receptor-bearing cells demonstrated weaker binding of APRIL, intermediate between cells expressing only TACI-S or TACI-L (Figure 4E). We also transfected TACI-S HEK-293T cells with increasing amounts of TACI-L (ratios of 1:1, 5:1, or 10:1) to further examine if adding TACI-L diluted ligand binding. As expected, increasing the TACI-L content again led to diminished binding of APRIL (Figure 4F).

The functional relevance of how receptor content, and thus ligand binding might affect receptor function, was then examined by luciferase induction in HEK-293T cells bearing either TACI-S or TACI-L, or for comparison, cells transfected with both constructs. NF-kB luciferase induction was again higher for TACI-S as compared to TACI-L cells, but intermediate for cells transfected with both constructs (Figure 4G). Thus, the relative content of TACI isoforms, with more or less of the short or long isoform, appears to regulate receptor signaling strength and therefore may permit modulation of B cell activation responses, and ultimately, cell differentiation.

### Both TACI isoforms co-localize with MyD88, TRAF6 in the endosomal compartment

**Figure 5:**
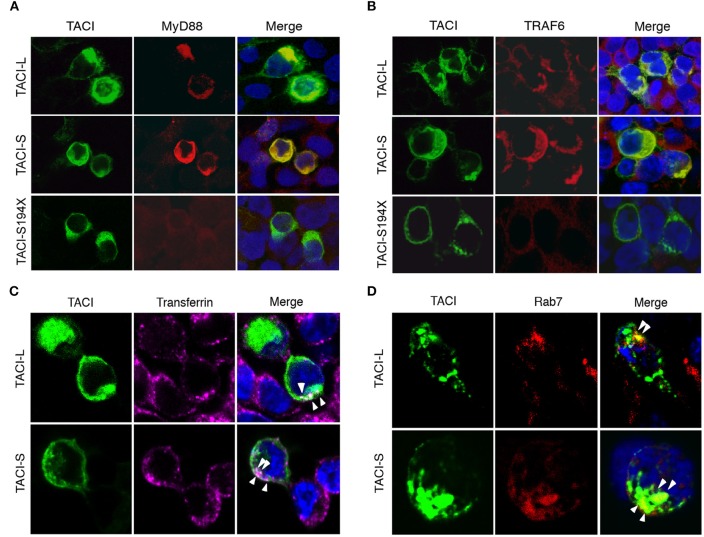
TACI co-localizes with MyD88 and TRAF6 in the late endosomal compartment. **(A)** mCherry labeled TACI-S, TACI-L or TACI-S194X isoform transfected HEK-293T cells, were stained with rabbit Ab to MyD88 or TRAF6; nuclei were stained with DAPI. Merged images show that each TACI isoform co-stains with MyD88 **(A)** and TRAF6 **(B)**, but colocalization is absent for the S194X mutant. **(C)** HEK-293T cells transfected with mCherry labeled TACI-L and TACI-S were incubated with Alexa Fluor 647-conjugated transferrin (40 μg/ml Tfn-647) at 37°C for 5 min fixed with 4% paraformaldehyde. Merged images show that both TACI-L and TACI-S co-localize with Tfn-647 (white arrows). **(D)** In other experiments, transfected HEK-293T cells were stained with mAb Rab7 as a marker of late endosomes, also showing co-localization. Samples were examined by Leica SP5 DMI confocal microscopy, acquiring 3 different xy planes with 63×/1.4 NA objective lenses (Carl Zeiss) with optimal z spacing (~0.016 μm). Images were processed using Adobe Photoshop.

As TACI is known to exhibit substantial intracellular expression in B cells (9, 19, 20), we also examined the intracellular localization of TACI-S and TACI-L isoforms in HEK-293T or TACI-transduced BJAB cells by confocal microscopy. We found that both isoforms co-localized with cytoplasmic MyD88, whereas the S194X mutant, lacking the TACI cytoplasmic sequence 228-233 (as expected from our previous work) failed to recruit MyD88 (Figure 5A and Supplemental Figure S2). As TRAF-6 engages the adjacent TACI PVE sequence (226–228) (26), we also sought binding of TACI isoforms with this adaptor protein. Both isoforms co-localized with TRAF6, but binding was again lost for the S194X variant, which has also lost the PVE motif (Figure 5B and Supplemental Figure S2). To further identify the intracellular location of TACI isoforms, we examined the uptake of labeled transferrin in HEK-293T cells transfected with either TACI-L or TACI-S (Figure 5C). Both isoforms were localized in the endocytic cytoplasmic region labeled by this marker. The intra-cytoplasmic localization of TACI isoforms, again similar, was further localized to the late endosomal compartment, as identified by Rab7 expression (Figure 5D).

### TACI isoforms co-localize and complex with TLR9

**Figure 6:**
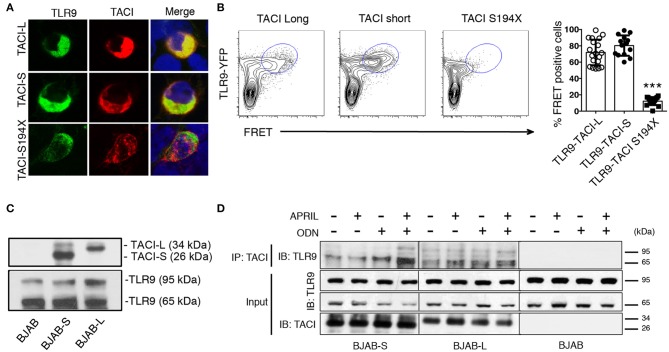
TACI isoforms are associated with activated cleaved TLR9. **(A)** HEK-293T cells were transfected with either mCherry labeled TACI-S or TACI-L or the S194X mutant (red) along with TLR9-YFP (green); cells were examined by confocal microscopy showing co-localization (yellow) in cells expressing TACI-L or –S, but absent in cells with the S194X mutant. **(B)** HEK-293T cells were transfected with either TACI-S, TACI-L, or the S194X mutant with TLR9-YFP, to determine complex formation using FRET using FACS (LSRFortessa) as described above. Data is representative of 10 experiments. Right panel shows the % of FRET positive cells in the double positive YFP and mCherry gate in these experiments. Scatter-plot graph shows the mean ± SD. ***p < 0.001, two-tailed paired Student *t*-test. **(C)** Lysates of parental BJAB cells or stably transduced with either TACI-S or TACI-L, were examined by immunoblotting using either anti-TACI (top) or anti-TLR9 antibody (bottom) after PAGE gel electrophoresis. Blotting shows both non-activated and activated TLR9 forms (95 and 65kDa) for all cells and TACI isoforms visible only for transduced cells. **(D)** In other experiments, TACI-S or TACI-L BJAB cells, were incubated for 48 h with APRIL, ODN, or both, were lysed and biotin-labeled goat anti-TACI complexes were collected with SA beads and immunoblotted after PAGE with anti-TLR9 antibody. The activated 65kDa TLR9 cleaved form was predominant in TACI-captured immune complexes (top panel). This effect was enhanced in BJAB-S cells on activation with ODN and even more with ODN + APRIL as activators. Lower panels show TACI and TLR9 expression for the input of these experiments.

Previously we showed that TACI is associated with the activated, cleaved form of TLR9 in human splenic B cells (9, 21). As TLR9 is cleaved in the endosome, and only the processed form recruits MyD88 (27), we used confocal microscopy to determine if TACI-S and/or TACI-L might merge with TLR9 in transfected HEK-293T cells (Figure 6A). Interestingly both TACI isoforms were capable of interacting with TLR9 in FRET experiments, again depending on the intracellular domain distal to aa194, as the FRET signals was abolished in the S194X mutant (Figure 6B). To validate the TACI intersection with TLR9 seen in these cells, we also examined the intersection between TLR9 and TACI isoforms in transduced BJAB cells, as these cells endogenously contain both TLR9 and MyD88 (28, 29). Transduced BJAB cells bore the respective isoform (but not native BJAB cells) and TLR9 in both the uncleaved (95 kDa) and the cleaved (65 kDa) forms (Figure 6C). However, when TLR9 was co-precipitated with a polyclonal anti-TACI antibody, the majority of TLR9 identified was the activated, cleaved form of (65 kDa) in these cells, as we showed previously for splenic B cells (9). Activation with ODN or ODN+APRIL, also increased the association of the activated form with TACI, as demonstrated in immunoblots (Figure 6D).

### Production of mAb to TACI isoforms and examination of human B cell populations

**Figure 7:**
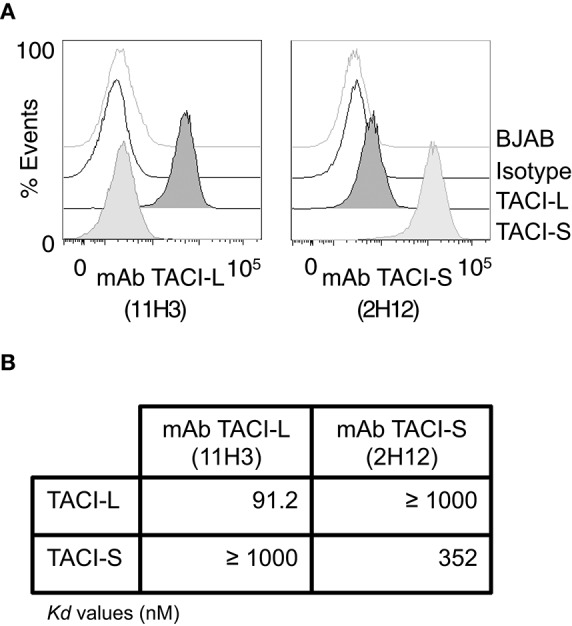
Monoclonal Antibodies to TACI isoforms **(A)** representative FACS (LSRII) histograms showing the specificity of the mAbs to TACI-L and TACI-S, tested on BJAB-L and BJAB-S cells along with control isotype and un-transduced BJAB cells. TACI-L is identified by mAb clone 11H3 and TACI-S by mAb 2H12. **(B)** The binding affinity of t mAb to TACI-L and TACI-S isoforms was determined by Microscale thermophoresis (MST), showing the specificity of each antibody. The change in the thermophoretic signal produces *K*d values (nM); lower indicating greater affinity. Data represent the mean and SD of three independent thermophoresis measurements.

To examine surface and intracellular expression of TACI isoforms in human B cell populations, we first raised a panel of mAbs to TACI-S. Clone 2H12 was chosen, as this mAb demonstrated staining of only TACI-L on BJAB cells, and not TACI-S. For TACI-L we used mAb clone 11H3 (Biosciences), as it possesses specificity only for the longer isoform (Figure 7A). The specificity of these clones for each isoform was also demonstrated by the MST assay, which showed the very different affinity of each mAb for these TACI isoform proteins (Figure 7B).

### Isoform expression in human B cell populations

**Figure 8:**
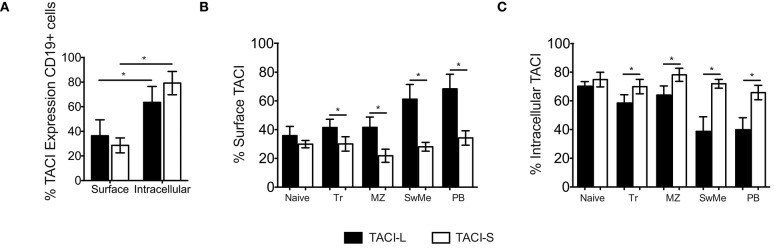
**(A)** TACI isoforms are differentially expressed in B cell subpopulations. Surface and intracellular TACI isoforms frequency in freshly isolated peripheral blood CD19+ cells. Data shows the mean ± SEM of 4 independent experiments for 4 normal donors. *p < 0.05, two-tailed paired Student *t*-test. **(B)** Percent of expression of surface and intracellular **(C)** TACI isoforms in different B cell subpopulations isolated from blood of 4 donors: Naïve (IgD+CD27-), Transitional (Tr) (IgM+CD38+), Marginal Zone (MZ) (IgD+CD27+), Switched Memory (SwMe) (IgD-CD27+), and Plasmablasts (PB) (IgD-CD27^hi^CD38^hi^). Data compile the results for 4 experiments. Data shows the mean ± SEM. *p < 0.05, two-tailed paired Student *t*-test.

While the surface expression of TACI on human B cells is well known, based on our confocal studies and the signaling requirements of this receptor, substantial intracellular TACI was also expected. Thus we used the above monoclonal antibodies to examine any differences in expression of isoforms on cell surface and in the intracellular compartment. As staining of permeabilized cells includes fluorescent contributions from cell surface staining, we determined the separate surface and intracellular expression for TACI-S and TACI-L, using a previously described method (22). While TACI-L was predominant as a surface receptor surface over TACI-S on human B cells, significantly more TACI-S was noted in intracellular compartment (*p* < 0.05) (Figure 8A). To dissect this further, we then examined human B cell populations in mononuclear cells from peripheral blood separately (Figures 8B,C and Supplemental Table SIII). While TACI-L was again more predominant as a surface receptor for transitional, marginal zone-like, switched memory B cells and plasmablasts, TACI-S was significantly more abundant in the intracellular cytoplasm, with especially increased expression in marginal zone and isotype switched (*p* < 0.05) and plasmablast B cells (*p* < 0.01).

### Effect of activation in isoform expression

**Figure 9:**
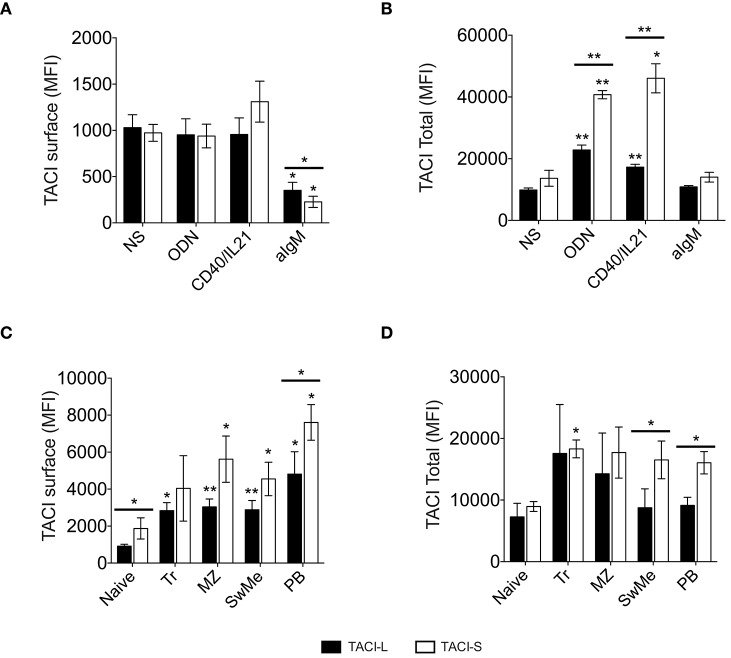
TACI isoforms expression in activated B cells. TACI isoform expression in non-permeabilized **(A)** and permeabilized **(B)** peripheral blood B cells from normal donors, cultured for 4 days in the presence or absence of ODN, CD40L/IL21, or anti-IgM. Data shows the mean ± SEM of 4 independent experiments. **(C,D)** TACI isoform expression was analyzed in B cell subpopulations isolated from freshly-isolated tonsils (*n* = 4): Naïve (IgD+CD27-), Transitional (Tr) (IgM+CD38+), Marginal Zone (MZ) (IgD+CD27+), Switched Memory (SwMe) (IgD-CD27+), and Plasmablasts (PB) (IgD-CD27^hi^CD38^hi^). Non permeabilized **(C)** and permeabilized cells **(D)** were examined. Data shows the mean ± SEM. *p < 0.05, **p < 0.01, two-tailed paired Student *t*-test.

To analyze the effect of B cell activation on the expression of TACI isoforms, peripheral blood mononuclear cells (PBMCs) of normal donors were stimulated with selected B cell activators: ODN, CD40L/IL21, or anti-IgM. After 4 days of incubation, while IgM stimulation had a somewhat decreased expression of both isoforms in non-permeabilized B cells (Figure 9A), cells activated with ODN or CD40L/IL21 showed a significant increase in total TACI expression, and especially TACI-S, as compared to the non-stimulated (NS) condition (Figure 9B). As B cells in lymphoid organs present a more activated state than in peripheral blood, we then analyzed the expression of these isoforms in B cell subpopulations from freshly isolated tonsils. These cells expressed more surface TACI-S in all populations and again, increased TACI-S in transitional, marginal, switched memory and plasmablasts (Figures 9C,D).

## Discussion

TACI is a complex B cell receptor that promotes T cell dependent and independent antibody secretion and plasma cell differentiation (5, 30). In humans, deficient B cell TACI expression, in either mutants or states of hemizygosity, interferes with B cell activation, antibody production and the development of memory and plasma cells (6, 7, 10). Since TACI–/– mice have an expanded B-cell pool with autoimmune manifestations (1, 31), and T cell-independent autoimmunity in a murine BAFF over-expression system depended on TACI, this receptor has been viewed as exerting tolerogenic functions (32). The negative regulatory role of TACI has also been evident in human immune deficiency, as patients with CVID who carry heterozygous *TNFRSF13B* mutations are more likely to have lymphoid hyperplasia and autoimmunity (9, 11, 33). TACI's potential tolerogenic mechanisms include inhibition of B cell proliferation, limiting clonal expansion, and promotion of B cell apoptosis (34, 35). We previously showed that TACI plays an essential role in establishing central B cell tolerance by preventing the production of autoreactive B cells in the bone marrow (9).

Many studies analyzing TACI function have been performed using murine models. However, the human *TNFRSF13B* gene contains an additional 5′ exon as compared to its murine counterpart. This allows differential RNA processing, leading to the production of two human isoforms (12), the roles of which have been unclear. The molecular mechanisms that regulate TACI splicing and by which TACI-S mediated its strong B cell activation remained to be determined; however, alternative mRNA splicing of other NF-kB signaling receptor genes (such as FAS, CD40) has been shown to control lymphoid growth and differentiation by generating contradictory signals (36–39). Our previous study on TACI isoforms revealed that the TACI short variant, which lacks the CRD1 domain, was indeed more efficient at inducing NF-kB activation, nuclear p65 translocation and plasma cell differentiation, as compared to the long isoform (13). However, differences in the ligand binding capacities of the two TACI isoforms were previously suggested using plasmon resonance (24).

We demonstrate here that the short TACI isoform displays clearly greater affinity for ligands BAFF and APRIL as compared to the long isoform. TACI-S also mediates markedly enhanced NF-kB activation, with or even without the addition of ligands BAFF or APRIL, a feature previously noted for this receptor (13, 15, 20). Using microscale thermophoresis, a cell-free system, we again validated that TACI-L protein displays a much weaker affinity for both BAFF and APRIL than TACI-S. As a result, the fully functional human TACI-S receptor appears more similar to the murine counterpart, supporting the previous suggestion that the CRD1 domain resembles an evolutionary remnant (24).

Interestingly, our FRET analysis revealed that cells could display hybrid TACI receptors, containing both isoforms. Further examining this possibility, we found that cells transfected or transduced with both TACI-S and TACI-L demonstrated both intermediate ligand binding and luciferase induction, as compared to cells expressing either isoform alone. As this may be true of human B cells, we suggest that the differential expression of either or both TACI isoforms could provide fine-tuning or tolerogenic differentiation responses for these cells. Since TACI-S more strongly mediates plasma cell differentiation, we postulate that B cells may express predominantly TACI-L during early B cell development and proliferation, while TACI-S would be more characteristic of terminal B cell differentiation. In line with this, additional controls for the same TACI/BAFFR/BCMA receptor system are provided by alternative splicing of genes encoding the ligands BAFF and APRIL, which also leads to structural variants with either activating or inhibiting properties (40, 41).

Following ligand binding, TACI directly interacts with MyD88 leading to NF-kB activation, dependent on the intracellular adaptors IRAK1, IRAK4, and TRAF6 (20). Since TACI engagement in conjunction with TLR ligands activates class-switch recombination and leads to IgG responses for T-independent antigens (20), we also explored the intracellular associations of TACI isoforms with TLR9. In agreement with the substantial intracytoplasmic TACI expression, we show that both TACI isoforms co-localized with MyD88 and TRAF6 in the endosomal compartment, depending on retention of a specific intracytoplasmic TACI sequence (20). Our previous work showed that TACI could be co-immunoprecipitated with activated TLR7 and TLR9 from human B cells (9, 21). We now show that both TACI isoforms, which share the same intracellular sequence, also associate with TLR9, while the truncated S194X TACI mutant does not, showing that terminal TACI intracellular domain is also required for binding TLR9, as it is for MyD88 (20). Here also we show that TLR9 activation increases TACI-S expression. Although we previously described that signaling through TLR9 increases mRNA TACI-S expression in human B cells (13), we now show that activation with a TLR9 agonist, as well as CD40L/IL21, increase TACI-S protein expression. However, more studies would need to demonstrate that role these activation signals play in the splicing of TACI mRNA.

For peripheral human B cells, which bear both TACI-S and TACI-L (13), we used specific mAbs, and found that the surface and intracellular distribution of isoforms displayed almost opposing expression, with greater TACI-L surface expression in all resting cell populations but enhanced intracellular TACI-S, especially in marginal zone, isotype switched memory B cells and plasma cells, in keeping with the role of this isoform in terminal differentiation. This effect was also observed after B cell activation. For tonsillar B cells however, perhaps due to chronic exposure to oral antigens, TACI-S was the predominate surface and intracellular isoform expressed.

In summary, alternative splicing of the human TACI gene leads to the production of two isoforms with different ligand binding and activation capabilities in human cells. TACI ligands BAFF and APRIL preferentially bind the short isoform, which is also more efficient at inducing NF-kB activation, driving B cell maturation to plasma cell development. While both isoforms are found in human B cells and co-localize in the cytoplasm with MyD88, TRAF6, and TLR9, there is differential surface and intracellular expression of these isoforms in human B cell populations.

## Ethics statement

This study was carried out in accordance with the recommendations of Mount Sinai Medical Center. The protocol was approved by the Mount Sinai Medical Center. All subjects gave written informed consent in accordance with the Declaration of Helsinki.

## Author contributions

YG-C and CC-R contributed conception and design of the study and performed the statistical analysis. YG-C performed experiments. SA performed MST assay. LR performed some experiments. JC acquired some data. CC-R wrote the first draft of the manuscript. AT, EM, and AC contributed with the discussion of the data. All authors contributed to manuscript revision, read, and approved the submitted version. YG-C and CC-R conceived the studies. CC-R obtained funding, provided study oversight, and drafted this manuscript.

### Conflict of interest statement

The authors declare that the research was conducted in the absence of any commercial or financial relationships that could be construed as a potential conflict of interest.

## References

[1] von Bulow GU van Deursen JM Bram RJ. Regulation of the T-independent humoral response by TACI. Immunity (2001) 14:573–82. 10.1016/S1074-7613(01)00130-3 11371359

[2] Castigli E Wilson SA Garibyan L Rachid R Bonilla F Schneider L. TACI is mutant in common variable immunodeficiency and IgA deficiency. Nat Genet. (2005) 37:829–34. 10.1038/ng1601 16007086

[3] Mackay F Schneider P Rennert P Browning J. BAFF AND APRIL: a tutorial on B cell survival. Ann Rev Immunol. (2003) 21:231–64. 10.1146/annurev.immunol.21.120601.141152 12427767

[4] Tsuji S Cortesao C Bram RJ Platt JL Cascalho M. TACI deficiency impairs sustained Blimp-1 expression in B cells decreasing long-lived plasma cells in the bone marrow. Blood (2011) 118:5832–9. 10.1182/blood-2011-05-353961 21984806 PMC3228499

[5] Mantchev GT Cortesao CS Rebrovich M Cascalho M Bram RJ. TACI is required for efficient plasma cell differentiation in response to T-independent type 2 antigens. J Immunol. (2007) 179:2282–8. 10.4049/jimmunol.179.4.2282 17675489

[6] Chinen J Martinez-Gallo M Gu W Cols M Cerutti A Radigan L. Transmembrane activator and CAML interactor (TACI) haploinsufficiency results in B-cell dysfunction in patients with Smith-Magenis syndrome. J Allergy Clin Immunol. (2011) 127:1579–86. 10.1016/j.jaci.2011.02.046 21514638 PMC3428026

[7] Romberg N Virdee M Chamberlain N Oe T Schickel JN Perkins T. TNF receptor superfamily member 13b (TNFRSF13B) hemizygosity reveals transmembrane activator and CAML interactor haploinsufficiency at later stages of B-cell development. J Allergy Clin Immun. (2015) 136:1315–25. 10.1016/j.jaci.2015.05.012 26100089 PMC4641026

[8] Salzer U Chapel HM Webster AD Pan-Hammarstrom Q Schmitt-Graeff A Schlesier M. Mutations in TNFRSF13B encoding TACI are associated with common variable immunodeficiency in humans. Nat Genet. (2005) 37:820–8. 10.1038/ng1600 16007087

[9] Romberg N Chamberlain N Saadoun D Gentile M Kinnunen T Ng YS. CVID-associated TACI mutations affect autoreactive B cell selection and activation. J Clin Invest. (2013) 123:4283–93. 10.1172/JCI69854 24051380 PMC3786721

[10] Martinez-Gallo M Radigan L Almejun MB Martinez-Pomar N Matamoros N Cunningham-Rundles C. TACI mutations and impaired B-cell function in subjects with CVID and healthy heterozygotes. J Allergy Clin Immunol. (2013) 131:468–76. 10.1016/j.jaci.2012.10.029 23237420 PMC3646641

[11] Zhang L Radigan L Salzer U Behrens TW Grimbacher B Diaz G. Transmembrane activator and calcium-modulating cyclophilin ligand interactor mutations in common variable immunodeficiency: clinical and immunologic outcomes in heterozygotes. J Allergy Clin Immunol. (2007) 120:1178–85. 10.1016/j.jaci.2007.10.001 17983875 PMC2908504

[12] Bossen C Schneider P. BAFF, APRIL and their receptors: structure, function and signaling. Seminars Immunol. (2006) 18:263–75. 10.1016/j.smim.2006.04.006 16914324

[13] Garcia-Carmona Y Cols M Ting AT Radigan L Yuk FJ Zhang L. Differential induction of plasma cells by isoforms of human TACI. Blood (2015) 125:1749–58. 10.1182/blood-2014-05-575845 25631768 PMC4357582

[14] Moreno JL Muguruza C Umali A Mortillo S Holloway T Pilar-Cuellar F. Identification of three residues essential for 5-hydroxytryptamine 2A-metabotropic glutamate 2 (5-HT2A.mGlu2) receptor heteromerization and its psychoactive behavioral function. J Biol Chem. (2012) 287:44301–19. 10.1074/jbc.M112.413161 23129762 PMC3531745

[15] Garibyan L Lobito AA Siegel RM Call ME Wucherpfennig KW Geha RS. Dominant-negative effect of the heterozygous C104R TACI mutation in common variable immunodeficiency (CVID). J Clin Invest. (2007) 117:1550–7. 10.1172/JCI31023 17492055 PMC1865037

[16] Fried AJ Rauter I Dillon SR Jabara HH Geha RS. Functional analysis of transmembrane activator and calcium-modulating cyclophilin ligand interactor (TACI) mutations associated with common variable immunodeficiency. J Allergy Clin Immunol. (2011) 128:226–8.e1. 10.1016/j.jaci.2011.01.048 21419480 PMC3121922

[17] Thompson JS Bixler SA Qian F Vora K Scott ML Cachero TG. BAFF-R, a newly identified TNF receptor that specifically interacts with BAFF. Science (2001) 293:2108–11. 10.1126/science.1061965 11509692

[18] Bossen C Cachero TG Tardivel A Ingold K Willen L Dobles M. TACI, unlike BAFF-R, is solely activated by oligomeric BAFF and APRIL to support survival of activated B cells and plasmablasts. Blood (2008) 111:1004–12. 10.1182/blood-2007-09-110874 17942754

[19] Chiu A Xu W He B Dillon SR Gross JA Sievers E. Hodgkin lymphoma cells express TACI and BCMA receptors and generate survival and proliferation signals in response to BAFF and APRIL. Blood (2007) 109:729–39. 10.1182/blood-2006-04-015958 16960154 PMC1785096

[20] He B Santamaria R Xu W Cols M Chen K Puga I. The transmembrane activator TACI triggers immunoglobulin class switching by activating B cells through the adaptor MyD88. Nat Immunol. (2010) 11:836–45. 10.1038/ni.1914 20676093 PMC3047500

[21] Sintes J Gentile M Zhang S Garcia-Carmona Y Magri G Cassis L. mTOR intersects antibody-inducing signals from TACI in marginal zone B cells. Nat Commun. (2017) 8:1462. 10.1038/s41467-017-01602-4 29133782 PMC5684130

[22] Hurt CM Ho VK Angelotti T. Systematic and quantitative analysis of G protein-coupled receptor trafficking motifs. Methods Enzymol (2013) 521:171–87. 10.1016/B978-0-12-391862-8.00009-0 23351739 PMC4024061

[23] Lee JJ Jabara HH Garibyan L Rauter I Sannikova T Dillon SR. The C104R mutant impairs the function of transmembrane activator and calcium modulator and cyclophilin ligand interactor (TACI) through haploinsufficiency. J Allergy Clin Immunol. (2010) 126:1234–41.e2. 10.1016/j.jaci.2010.08.017 20889194 PMC3122265

[24] Hymowitz SG Patel DR Wallweber HJ Runyon S Yan M Yin J. Structures of APRIL-receptor complexes: like BCMA, TACI employs only a single cysteine-rich domain for high affinity ligand binding. J Biol Chem. (2005) 280:7218–27. 10.1074/jbc.M411714200 15542592

[25] Wienken CJ Baaske P Rothbauer U Braun D Duhr S. Protein-binding assays in biological liquids using microscale thermophoresis. Nat Commun. (2010) 1:100. 10.1038/ncomms1093 20981028

[26] Xia XZ Treanor J Senaldi G Khare SD Boone T Kelley M. TACI is a TRAF-interacting receptor for TALL-1, a tumor necrosis factor family member involved in B cell regulation. J Exp Med. (2000) 192:137–43. 10.1084/jem.192.1.137 10880535 PMC1887716

[27] Ewald SE Lee BL Lau L Wickliffe KE Shi GP Chapman HA. The ectodomain of Toll-like receptor 9 is cleaved to generate a functional receptor. Nature (2008) 456:658–62. 10.1038/nature07405 18820679 PMC2596276

[28] Chockalingam A Brooks JC Cameron JL Blum LK Leifer CA. TLR9 traffics through the Golgi complex to localize to endolysosomes and respond to CpG DNA. Immunol Cell Biol. (2009) 87:209–17. 10.1038/icb.2008.101 19079358 PMC2753824

[29] Ressing ME Keating SE van Leeuwen D Koppers-Lalic D Pappworth IY Wiertz EJ. Impaired transporter associated with antigen processing-dependent peptide transport during productive EBV infection. J Immunol. (2005) 174:6829–38. 10.4049/jimmunol.174.11.6829 15905524

[30] Castigli E Wilson SA Elkhal A Ozcan E Garibyan L Geha RS. Transmembrane activator and calcium modulator and cyclophilin ligand interactor enhances CD40-driven plasma cell differentiation. J Allergy Clin Immunol. (2007) 120:885–91. 10.1016/j.jaci.2007.06.012 17689597 PMC2121612

[31] Seshasayee D Valdez P Yan M Dixit VM Tumas D Grewal IS. Loss of TACI causes fatal lymphoproliferation and autoimmunity, establishing TACI as an inhibitory BLyS receptor. Immunity (2003) 18:279–88. 10.1016/S1074-7613(03)00025-6 12594954

[32] Jacobs HM Thouvenel CD Leach S Arkatkar T Metzler G Scharping NE. Cutting edge: BAFF promotes autoantibody production via TACI-dependent activation of transitional B cells. J Immunol. (2016) 196:3525–31. 10.4049/jimmunol.1600017 27022196 PMC4868625

[33] Salzer U Bacchelli C Buckridge S Pan-Hammarstrom Q Jennings S Lougaris V. Relevance of biallelic versus monoallelic TNFRSF13B mutations in distinguishing disease-causing from risk-increasing TNFRSF13B variants in antibody deficiency syndromes. Blood (2009) 113:1967–76. 10.1182/blood-2008-02-141937 18981294 PMC2651012

[34] Tsuji S Stein L Kamada N Nunez G Bram R Vallance BA. TACI deficiency enhances antibody avidity and clearance of an intestinal pathogen. J Clin Invest. (2014) 124:4857–66. 10.1172/JCI74428 25271628 PMC4347253

[35] Kanno Y Sakurai D Hase H Kojima H Kobata T. TACI induces cIAP1-mediated ubiquitination of NIK by TRAF2 and TANK to limit non-canonical NF-kappaB signaling. J Recept Signal Transduct Res. (2010) 30:121–32. 10.3109/10799891003634509 20184394

[36] Cheng J Zhou T Liu C Shapiro JP Brauer MJ Kiefer MC. Protection from Fas-mediated apoptosis by a soluble form of the Fas molecule. Science (1994) 263:1759–62. 10.1126/science.7510905 7510905

[37] Razani B Reichardt AD Cheng G. Non-canonical NF-kappaB signaling activation and regulation: principles and perspectives. Immunol Rev. (2011) 244:44–54. 10.1111/j.1600-065X.2011.01059.x 22017430

[38] Tone M Tone Y Fairchild PJ Wykes M Waldmann H. Regulation of CD40 function by its isoforms generated through alternative splicing. Proc Natl Acad Sci USA. (2001) 98:1751–6. 10.1073/pnas.98.4.1751 11172023 PMC29329

[39] Ergun A Doran G Costello JC Paik HH Collins JJ Mathis D. Differential splicing across immune system lineages. Proc Natl Acad Sci USA. (2013) 110:14324–9. 10.1073/pnas.1311839110 23934048 PMC3761616

[40] Gavin AL Ait-Azzouzene D Ware CF Nemazee D. DeltaBAFF, an alternate splice isoform that regulates receptor binding and biopresentation of the B cell survival cytokine, BAFF. J Biol Chem. (2003) 278:38220–8. 10.1074/jbc.M306852200 12867412 PMC3792716

[41] Furuya T Koga M Hikami K Kawasaki A Tsuchiya N. Effects of APRIL (TNFSF13) polymorphisms and splicing isoforms on the secretion of soluble APRIL. Modern Rheumatol. (2012) 22:541–9. 10.3109/s10165-011-0539-z 21984075

