# Corrigendum: TACI Isoforms Regulate Ligand Binding and Receptor Function

**DOI:** 10.3389/fimmu.2019.02772

**Published:** 2019-12-03

**Authors:** Yolanda Garcia-Carmona, Adrian T. Ting, Lin Radigan, Sai Krishna Athuluri Divakar, Jose Chavez, Eric Meffre, Andrea Cerutti, Charlotte Cunningham-Rundles

**Affiliations:** ^1^Department of Clinical Immunology, Precision Immunology Institute, Icahn School of Medicine at Mount Sinai, New York, NY, United States; ^2^Department of Oncological Sciences, Icahn School of Medicine at Mount Sinai, New York, NY, United States; ^3^Department of Immunobiology, Yale University School of Medicine, New Haven, CT, United States; ^4^Catalan Institute for Research and Advance Studies (ICREA), Barcelona, Spain; ^5^Program for Inflammatory and Cardiovascular Disorders, Institut Hospital del Mar d'Investigacions Mèdiques (IMIM), Barcelona, Spain; ^6^Department of Medicine and Pediatrics, Icahn School of Medicine at Mount Sinai, New York, NY, United States

**Keywords:** TACI, isoforms, B cell, activation, TLR9

In the original article, there was a mistake in Figure 1A and Figure 2A as published. In Figure 1A, two panels of the 12 panels were mistakenly duplicated; in Figure 2A, one panel of the 12 panels was also duplicated. The numbers given are correct. The corrected Figure 1, Figure 2 and legends appear below.

**Figure 1:**
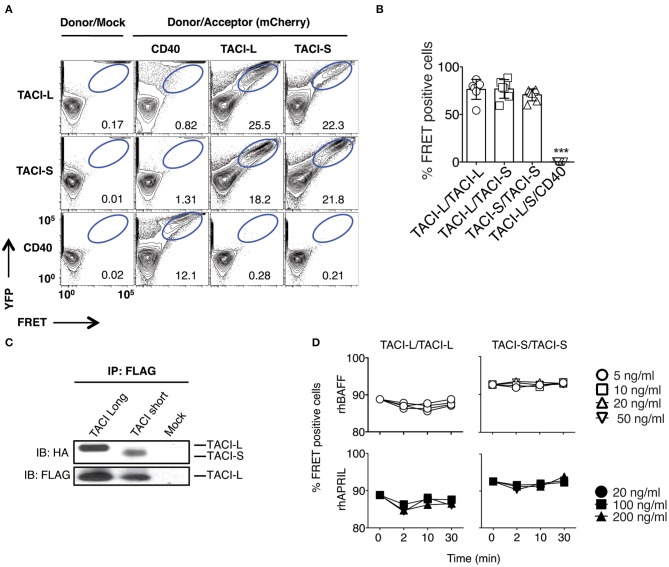
TACI Isoforms form hybrid complexes detected by FRET. **(A)** To examine isoform complexes, YFP and mCherry labeled TACI-L, TACI-S, and/or CD40-eYFP as a control, were co-transfected into HEK-293T cells. After 48 h, the molecular association was analyzed by FRET using FACS (LSRII). A minimum of 50,000 positive cells were examined in all experiments. The dot plot results in each panel, are shown for one sample, representative of the results for 6 different experiments. The numerical data given in each panel, are the percent of FRET positive cells, of the live cells in the gate, averaged for all 6 experiments. **(B)** Frequency of FRET positive cells in the double positive YFP and mCherry gate from **(A)**. Graph shows the mean ± SD. ***p < 0.001, two-tailed paired Student *t*-test of 6 independent experiments. **(C)** Either HA-labeled TACI-S or TACI-L transfected into HEK-293T cells, could be precipitated with TACI-L-FLAG as demonstrated in immuno-blotting using anti-HA staining. Lower panel shows FLAG expression as control. **(D)** Addition of increasing amounts of ligands BAFF or APRIL to the TACI-S and TACI-L complexes had no effect on the FRET signal, indicating that receptor assembly in these cells is ligand independent. Data are the mean ± SD from 3 independent experiments.

**Figure 2:**
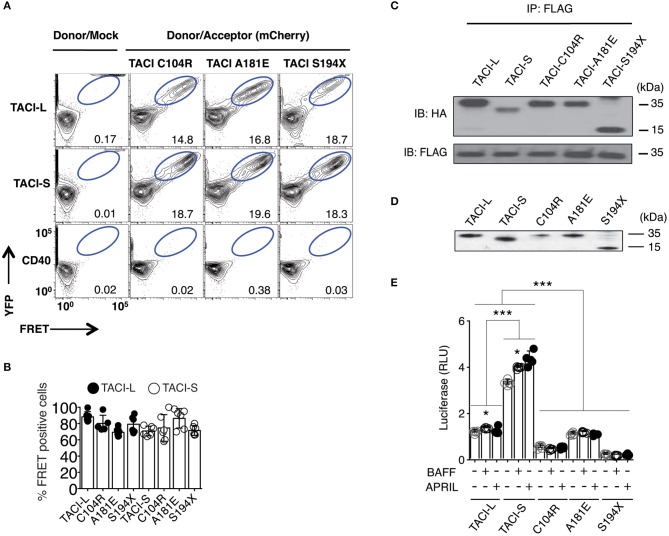
TACI variants with missense mutations bind un-mutated isoforms but lack signaling function. **(A)** TACI mCherry labeled mutants, C104R, A181E, and S194X were generated in the TACI-L and TACI-S isoforms, and co-transfected into HEK**-**293T cells with WT TACI-YFP. These were examined in FRET experiments by FACS (LSRII) to judge complex formation using CD40-eYFP as a control. The dot plot results in each panel, are shown for one sample, representative of the results for 6 different experiments. The numerical data given in each panel, are the percent of FRET positive cells, of the live cells in the gate, averaged for all 6 experiments. **(B)** Frequency of FRET positive cells in the double positive YFP and mCherry gate. Data shows average ± SD from 6 independent experiments. In other experiments, ligands, APRIL, or BAFF (0, 5, 20, or 50 ng/ml) were added to judge the effects on FRET signal, showing no alteration in the signal (not shown). **(C)** For validation of complexes found in FRET, complexes forming with FLAG-TACI were precipitated with anti-FLAG sepharose beads and run on 10% PAGE gels; immunoblots were developed with an anti-HA antibody. Lower panel shows FLAG expression control. **(D)** To examine NF-kB luciferase induction, TACI-S, TACI-L or mutant C104R, A181E and S194X constructs were transfected into HEK-293T cells, along with NF-kB–luc reporter and control pRL-null plasmids and cultured for 48 h; TACI expression was confirmed by western blot. **(E)** These cells were cultured with or without activation for 6 h with 100 ng/ml APRIL or BAFF. Reporter gene activity was determined, and NF-kB luciferase induction normalized to Renilla luciferase. Values reported are represented as Relative Luciferase Activity (RLA) and are the mean ± SD from 5 to 7 independent experiments. *p < 0.05; ***p < 0.001, two-tailed paired Student *t*-test. Western blot shows that all constructions are expressed.

The authors apologize for this error and state that this does not change the scientific conclusions of the article in any way. The original article has been updated.

