# Supplementary material for: TACI Isoforms Regulate Ligand Binding and Receptor Function
Source: Front Immunol. 2018 Oct 2;9:2125. doi: 10.3389/fimmu.2018.02125 (PMC6176016; doi:10.3389/fimmu.2018.02125)
Supplement: Supplementary file 1 [file Table_1.DOCX]

**Supplemental Table SI. PCR and Site-Directed Mutagenesis Primers**

| TACI *Xho*I Fwd | 5’-GGCTCGAGATGAGTGGCCTGG-3′ |
| --- | --- |
| TACI *Hind*III Rev | 5’-CCAGTCAAGCTTTCTGCACCTGGGCC-3′ |
| mCherry *Hind*III Fwd | 5’-GGTAAGCTTGATGGTGAGCAAGG-3′ |
| Cherry *Not*I Rev | 5’-CAAGCGGCCGCCTACTTGTACAG-3′ |
| eYFP *Hind*III Fwd | 5′-AGGATGGCTCATTCAAAGCACGGTCTAAAAGAAGAAATGACAATG-3′ |
| eYFP *Not*I Rev | 5′- GAAGACGGAACCGGACTTCGCCGGCGAAG -3′ |
| TACI C104R Fwd | 5’-GTGCATACTTC**CGT**GAGAACAAGCT-3′ |
| TACI C104R Rev | 5’-CACGTATGAAG**ACG**CTCTTGTTCGA-3′ |
| TACI A181E Fwd | 5’-CTGCTTCCTGGTG**GAG**GTGGCCTGCTTCCTC-3′ |
| TACI A181E Rev | 5’-GACGAAGGACCAC**CTC**CACCGGACGAAGGAG-3′ |
| TACI S194X Fwd | 5’-GGATCCCTGC**TAA**TGCCAGCCCCGCTCAAG-3′ |
| TACI S194X Rev | 5’-CCTAGGGACG**TTA**ACGGTCGGGGCGAGTTC-3′ |

* Bold letters show the mutation site
