# Supplementary material for: TACI Isoforms Regulate Ligand Binding and Receptor Function
Source: Front Immunol. 2018 Oct 2;9:2125. doi: 10.3389/fimmu.2018.02125 (PMC6176016; doi:10.3389/fimmu.2018.02125)
Supplement: Supplementary file 2 [file Table_2.DOCX]

**Supplemental Table SII. In-Fusion cloning Primers**

| pcDNA-HA-TACI Delta E1-CRD1 | PCR1 | S | 5’CTAAAGCTTGGCATGTACCCATACGATGTTCCCGATTACGCCCTCGAGATGTGGTCACTCAGCTGCCGCAAGGAG-3’ |
| --- | --- | --- | --- |
|  |  | AS | 5’-AATTGGCGGCCGCTTATGCACCTGGGCCCCCCTCCTGGG-3’ |
|  | PCR2 | S | 5’-TGGCTAGCGTTTAAACTTAAGCTTGGCATGTACCCATACGATGTTCC-3’ |
|  |  | AS | 5’-AATTGGCGGCCGCTTATGCACCTGGGCCCCCCTCCTGGG-3’ |
| pcDNA-HA-TACI Delta CRD2 | PCR1 | S | 5’- TGGCTAGCGTTTAAACTTAAGCTTGGCATGTACCCATACGATGTTCC-3’ |
|  |  | AS | 5’-CTTGTTCTCGCTGAGTGACCTGCAGAAGGCTGCACAG-3’ |
|  | PCR2 | S | 5’- CCTGTGCAGCCTTCTGCAGGTCACTCAGCGAGAACAAGCTCAGG-3’ |
|  |  | AS | 5’- CGGGCCCTCTAGACTCGAGCGGCCGCTTATGCACCTGGGCCCCCCT-3’ |
|  | PCR3 | S | 5’- TGGCTAGCGTTTAAACTTAAGCTTGGCATGTACCCATACGATGTTCC-3’ |
|  |  | AS | 5’-CGGGCCCTCTAGACTCGAGCGGCCGCTTATGCACCTGGGCCCCCCT-3’ |
| pcDNA-HA-TACI Delta 105-166 | PCR1 | S | 5’CTAAAGCTTGGCTACCCATACGATGTTCCCGATTACGCCCTCGAGATGAGTGGCCTGGGCCGGAGCAGGCGA-3’ |
|  |  | AS | 5’-GCAGAGCCCCAGCGTGCTACAGAAGTATGCACATTGC -3’ |
|  | PCR2 | S | 5’-CAATGTGCATACTTCTGTAGCACGCTGGGGCTCTGCCTGTGTGCCG -3’ |
|  |  | AS | 5’-CGGGCCCTCTAGACTCGAGCGGCCGCTTATGCACCTGGGCCCCCCT -3’ |
|  | PCR3 | S | 5’CTAAAGCTTGGCTACCCATACGATGTTCCCGATTACGCCCTCGAGATGAGTGGCCTGGGCCGGAGCAGGCGA-3’ |
|  |  | AS | 5’-CGGGCCCTCTAGACTCGAGCGGCCGCTTATGCACCTGGGCCCCCCT -3’ |
| pcDNA-HA-TACI Delta 21-104 | PCR1 | S | 5’- TGGCTAGCGTTTAAACTTAAGCTTGGCATGTACCCATACGATGTTCC-3’ |
|  |  | AS | 5’-GCTCCTGAGCTTGTTCTCGCGCTCCTCCTGGTCCAC -3’ |
|  | PCR2 | S | 5’GAGCCGTGTGGACCAGGAGGAGCGCGAGAACAAGCTCAGGAGCCCAGTGAACCTTCCACCAGAGC -3’ |
|  |  | AS | 5’-CGGGCCCTCTAGACTCGAGCGGCCGCTTATGCACCTGGGCCCCCCT -3’ |
|  | PCR3 | S | 5’- TGGCTAGCGTTTAAACTTAAGCTTGGCATGTACCCATACGATGTTCC-3’ |
|  |  | AS | 5’- CGGGCCCTCTAGACTCGAGCGGCCGCTTATGCACCTGGGCCCCCCT-3’ |
| pcDNA-FLAG-TACI | PCR1 | S | 5’GGAAGCTTCGACTACAAAGACGATGACGACAAGAAACTCGAGATGAGTGGCCTGGGCCGGAGCAGGCG-3’ |
|  |  | AS | 5’-GCGGCCGCTTATGCACCTGGGCCCCCCTCCTGGG-3’ |
| pcDNA-HA-TACI | PCR1 | S | 5’CTAAAGCTTGGCATGTACCCATACGATGTTCCCGATTACGCCCTCGAGATGAGTGGCCTGGGCCGGAGCAGGCGA-3’ |
|  |  | AS | 5’-CGGGCCCTCTAGACTCGAGCGGCCGCTTATGCACCTGGGCCCCCCT-3’ |
|  | PCR2 | S | 5’-TGGCTAGCGTTTAAACTTAAGCTTGGCATGTACCCATACGATGTTCC-3’ |
|  |  | AS | 5’-CGGGCCCTCTAGACTCGAGCGGCCGCTTATGCACCTGGGCCCCCCT-3’ |
