# Supplementary material for: TACI Isoforms Regulate Ligand Binding and Receptor Function
Source: Front Immunol. 2018 Oct 2;9:2125. doi: 10.3389/fimmu.2018.02125 (PMC6176016; doi:10.3389/fimmu.2018.02125)
Supplement: Supplementary file 3 [file Table_3.DOCX]

|  | | **% Surface expression (Mean±SEM)** | | **% Intracellular expression (Mean±SEM)** | |
| --- | --- | --- | --- | --- | --- |
|  | **% of cells *(n=4)*** | **TACI-L** | **TACI-S** | **TACI-L** | **TACI-S** |
| **Total B cells (CD19+)** | 8.44±3.1 (7.2-11.2) | 36.43±12 | 24.13±15 * | 63.56±14 | 75.83±5 * |
| **Naïve**  **(CD27-IgD+)** | 65.63±13.73 (58.0-72.1) | 35.82±12 | 29.91±4 | 70.21±5 | 74.83±10 |
| **Tr**  **(CD38+IgM+)** | 1.45±0.6 (1.0-3.6) | 41.48±9 | 30.09±8 * | 58.51±9 | 69.9±8 * |
| **MZ**  **(CD27+IgD+)** | 14.05±5.4 (13.4-21.4) | 41.58±14 | 21.85±9 * | 64.01±11 | 78.14±9 * |
| **SwMe (CD27+IgD-)** | 9.53±2.6 (9.2-18.9) | 61.26±17 | 28.11±5 * | 38.73±17 | 71.88±5 * |
| **PB (CD27^hi^CD38^hi^)** | 0.67±0.2 (0.6-1.6) | 68.39±20 | 34.23±10 ** | 39.88±14 | 65.76±10 ** |

**Supplemental Table SIII. Frequency of B cell subpopulations and average expression of TACI isoforms in human peripheral blood.** Flow cytometry (LSRFortessa) analysis of TACI isoforms expression in different B cell subpopulations (n=4), using specific monoclonal antibodies for each isoform. Frequency of total CD19+ B cells (as percentage of all lymphocytes) as well as B cell subsets (as percentage of all CD19+ cells) is shown as means±SD (upper line) and as the corresponding interquartile ranges (25^th^ and 75^th^ percentiles, lower line). Percentage of TACI isoforms surface and intracellular expression in CD19+ B cells and in different B cell subpopulations is shown as means±SD. The comparison between TACI-L and TACI-S expression in each compartment is also shown (* p<0.05, ** p<0.01, Two-tailed Paired t Student).
