# Supplementary material for: TACI Isoforms Regulate Ligand Binding and Receptor Function
Source: Front Immunol. 2018 Oct 2;9:2125. doi: 10.3389/fimmu.2018.02125 (PMC6176016; doi:10.3389/fimmu.2018.02125)
Supplement: Supplementary file 4 [file Presentation_1.PDF]

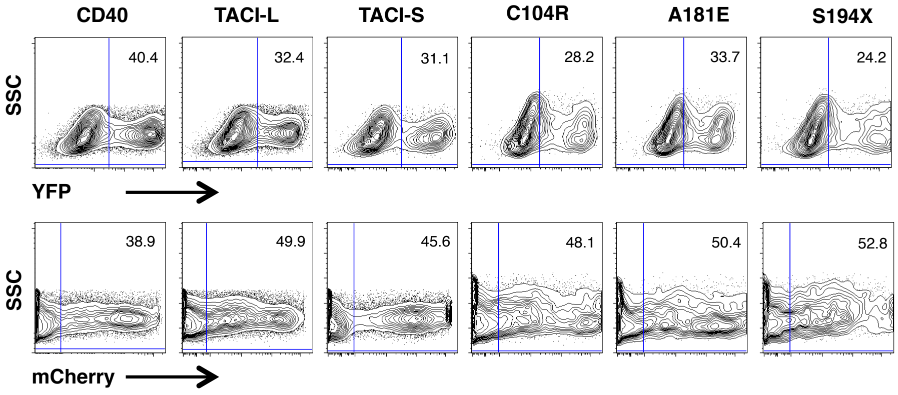

Supplemental Figure S1. YFP and mCherry expression in double-transfected cells for FRET experiments. Representative flow cytometry (LSRII) contour plots showing the expression of the reporter genes YFP and mCherry for Figure 1A and Figure 1B.
