# Supplementary material for: TACI Isoforms Regulate Ligand Binding and Receptor Function
Source: Front Immunol. 2018 Oct 2;9:2125. doi: 10.3389/fimmu.2018.02125 (PMC6176016; doi:10.3389/fimmu.2018.02125)
Supplement: Supplementary file 5 [file Data_Sheet_1.PDF]

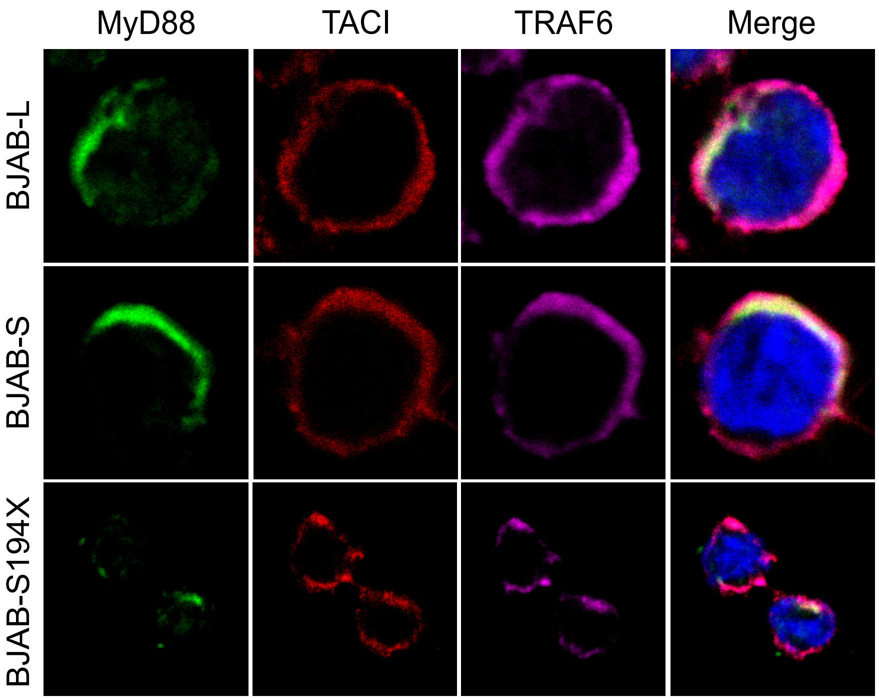

Supplemental Figure S2. TACI, MyD88 and TRAF6 expression in BJAB cells. BJAB-S, BJAB-L or BJAB-S194X isoform cells, were stained with MyD88 (green), TACI (red) and TRAF6 (purple); nuclei were stained with DAPI. Merged images show that each TACI isoform co-stains with MyD88 and TRAF6 but poorly for the S194X mutant. Samples were examined by Leica SP5 DMI confocal microscopy, acquiring 3 different xy planes with 63×/1.4 NA objective lenses (Carl Zeiss) with optimal z spacing (~0.016 µm). Images were processed using Adobe Photoshop.
